# Phenotypic Characterization of *Mycoplasma synoviae* Induced Changes in the Metabolic and Sensitivity Profile of *In Vitro* Infected Chicken Chondrocytes

**DOI:** 10.1155/2014/613730

**Published:** 2014-08-26

**Authors:** Daliborka Dušanić, Dušan Benčina, Mojca Narat, Irena Oven

**Affiliations:** Department of Animal Science, Biotechnical Faculty, University of Ljubljana, Groblje 3, 1230 Domzale, Slovenia

## Abstract

In infectious synovitis caused by* Mycoplasma synoviae* chicken chondrocytes (CCH) may come into direct contact with these bacteria that are also capable of invading CCH* in vitro*. In this study, phenotype microarrays were used to evaluate the influence of* Mycoplasma synoviae* on the global metabolic activity of CCH. Therefore, CCH were cultured in the presence of 504 individual compounds, spotted in wells of 11 phenotype microarrays for eukaryotic cells, and exposed to* Mycoplasma synoviae* membranes or viable* Mycoplasma synoviae. *Metabolic activity and sensitivity of normal cells versus infected cells were evaluated. Metabolic profiles of CCH treated with viable* Mycoplasma synoviae* or its membranes were significantly different from those of CCH alone. CCH treated with* Mycoplasma synoviae* membranes were able to use 48 carbon/nitrogen sources not used by CCH alone. Treatment also influenced ion uptake in CCH and intensified the sensitivity to 13 hormones, 5 immune mediators, and 29 cytotoxic chemicals. CCH were even more sensitive to hormones/immune mediators when exposed to viable* Mycoplasma synoviae*. Our results indicate that exposure to* Mycoplasma synoviae* or its membranes induces a wide range of metabolic and sensitivity modifications in CCH that can contribute to pathological processes in the development of infectious synovitis.

## 1. Introduction

*M. synoviae* is a major poultry pathogen causing respiratory and systemic disease, autoimmune disorders, and infectious synovitis in chickens and turkeys [1]. It has been detected in many internal organs, as well as in the synovial fluid and joint tissues of chickens with infectious synovitis [2–4].* M. synoviae* also invades nonphagocytic chicken cells* in vitro*, including chicken chondrocytes (CCH) [5]. In joints,* M. synoviae* and its membrane proteins may come into contact with CCH, as well as with immune cells including macrophages, T-lymphocytes, and B-lymphocytes causing their activation [1, 6]. It has been demonstrated that* M. synoviae, *particularly its membrane lipoprotein MSPB, induces NO, IL-1*β*, and IL-6 synthesis in chicken macrophages [7–9]. The presence of* M. synoviae* or its immunogenic proteins could have important impact on the host cell metabolic pathways and sensitivity to environmental conditions, including the host's immune molecules, and could influence the intensity of joint inflammation and local tissue destruction. The link between joint inflammation and joint destruction is poorly understood both in humans and animal models. Human chondrocytes respond to several immune mediators, including proinflammatory cytokines, chemokines, and nitric oxide, which probably act as a network [10–14]. The effect of immune mediators on chondrocytes of adult chickens with infectious synovitis has not yet been documented, although infectious synovitis could be considered as an animal model for studying bacteria-induced arthritic diseases.

Apart from induction of cytokines, bacterial infection could have a profound impact on the uptake of ions and carbon/nitrogen sources in infected cells. The influence of* Mycoplasma* infection on the metabolic level of eukaryotic cells has been documented only for* M. pneumoniae *[15] and for the most common cell-culture infectant,* M. hyorhinis* [16]. In this study, we present the first report of the influence of* M. synoviae* infection on chicken chondrocyte metabolism, as analysed by phenotype microarrays.

Phenotype microarrays (PM) have been developed in order to evaluate pathways that contribute to energy production and cell sensitivity to different environmental factors, including any kind of stress, including bacterial infection. Although most of the research using phenotype microarrays has been done to analyze the metabolic phenotype of prokaryotes, microarrays have also been developed for eukaryotic cells (PMM) [17]. In this study, we have used PM technology to evaluate the changes in physiology of CCH, infected with* M. synoviae *or its components, which were manifested as alterations in the level of respiration (energy production) in the presence of various compounds, many of which can be found in the complex environment of infectious synovitis.

## 2. Materials and Methods

### 2.1. Chicken Chondrocytes

Chicken chondrocytes (CCH, third passage), obtained as described previously [5], were cultured in Dulbecco's modified Eagle's medium supplemented with 2.5% chicken serum, 7.5% foetal bovine serum (FBS) (all from Sigma-Aldrich, Germany) and 1 *μ*g gentamicin/Ml. Cells were grown in culture flasks at 38°C in a 5% CO_2_ atmosphere.

### 2.2. *M. synoviae* Culture

Cultures of the* M. synoviae* type strain WVU 1853 were grown as described previously [1, 5]. The number of colony forming units was determined as described previously [18].

### 2.3. *M. synoviae* Membrane Fraction Preparation

For the preparation of* M. synoviae* membrane fraction, a modified protocol for osmotic lysis [19] was used. Briefly,* M. synoviae* WVU 1853 broth culture (~500 mL) in the late logarithmic phase of growth was pelleted, washed in 0.02 M Tris-HCl solution, and treated with 5 mL of 2 M glycerol for 10 min at 38°C. Cell suspension was rapidly injected into 50 mL of dH_2_O and incubated for 15 min at 38°C, and the membranes were collected by centrifugation (30 min at 34000 ×g). To exclude any viable* M. synoviae*, the membrane fraction was treated with phosphate buffered saline (PBS) containing 0.5 mg gentamicin/mL for 4 h at 38°C. The membrane fraction was collected by centrifugation and resuspended in 100 *μ*L of sterile PBS. Protein concentration in* M. synoviae* membrane fraction was determined by a modified Bradford assay [20].

### 2.4. Optimisation of PM Experiment Parameters

PMs use Biolog's patented redox chemistry, employing cell respiration as a universal reporter. The redox assay provides for both amplification and precise quantitation of phenotypes. Redox Dye Mix MA and MB each contain a water-soluble nontoxic tetrazolium reagent that can be used with virtually any type of animal cell line or primary cell, but some cells prefer to use MA dye and other MB dye, therefore it is essential to optimize dye selection with the cell line used in the experiment [17].

**Figure 1:**
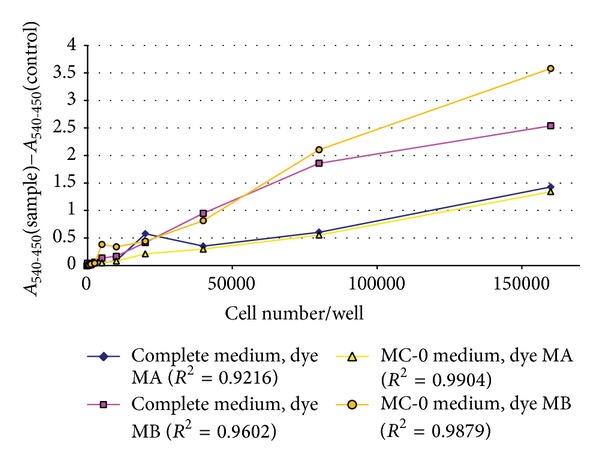
Optimization of formazan production by CCH. CCH were plated in complete medium or MC-0 medium at indicated number of cells per well and incubated with Biolog Redox dye mix MA or MB for 6 hours. The background signal (medium incubated with appropriate dye, but with no cells) was subtracted from the data.

In order to select the proper dye mix we performed optimisation experiment to determine (i) which of the two dyes (MA or MB) available for phenotype microarrays is more appropriate for CCH, (ii) the minimal number of CCH that generate a sufficient amount of formazan within two to six hours, (iii) the linear range of detection for absorbance measurements, and (iv) the amount of dye reduction in MC-0 after 2 days of incubation (background signal). CCH were detached from culture flasks using 0.05% trypsine-EDTA solution (Sigma-Aldrich, Germany) and centrifuged for 5 min at 300 ×g. Cell viability was 98% as assessed by trypan blue staining (Sigma-Aldrich, Germany). CCH were washed three times in PBS and resuspended in two different media. Complete medium was composed of IF-M1 medium (Biolog, US) lacking phenol red, glucose, and glutamine, supplemented with 3.75% FBS, 1.25% chicken serum (Sigma-Aldrich, Germany), 11 mM glucose, and 2 mM glutamine (Sigma-Aldrich, Germany). Medium MC-0 was composed of IF-M1 medium supplemented with 3.75% FBS, 1.25% chicken serum, and 0.3 mM glutamine. A 96-well cell culture plate was inoculated with CCH in the following way: two-fold serial dilutions of CCH ranging from 16 × 10^4^ to zero cells/well (total volume 100 *μ*L/well) were made both in complete medium (rows A to D) and MC-0 medium (rows E to H). The plate was incubated for 1 h at 38°C in a 5% CO_2_ atmosphere, after which 20 *μ*L of Biolog Redox dye MA was added to rows A and C while MB dye was added to rows B and D. A microplate reader was used to measure A_540_ and A_450_ every 15 min for the first two hours and then every half hour for the next 4 hours. The plate was incubated for additional 42 h, after which MA dye was added to rows C and G and MB dye to rows D and H and the absorbance was measured as before. Results confirmed that MB dye is metabolised more rapidly and generates a higher signal compared to MA dye in a given time period (Figure 1). Therefore, MB dye was used in subsequent experiments. The minimal number of CCH required to give detectable signals in the first two hours of incubation in complete medium with MB dye was 10^4^ cells (data not shown). Therefore, 10^4^ CCH/well was the optimal starting concentration of cells for metabolic plates (PMMs 1–4), while sensitivity plates (PMMs 5–8, 11, 13, and 14) required 2 × 10^4^ CCH/well for optimal signal detection. The kinetics of formazan production were in the linear range of detection for the entire 6-hour period of measurements. After two-day incubation in MC-0 medium, the amount of formazan product was low, thus providing high signal-to-background ratio.

### 2.5. PM Experiment Design

PMMs (Biolog, US) used in this study were PMMs 1–4 (precoated with carbon and nitrogen (C/N) sources), PMM 5 (ions), PMMs 6–8 (hormones and immune mediators), PMM 11, PMM 13, and PMM 14 (cytotoxic/anticancer drugs). A complete list of all compounds and their organisation on microplates is available at http://www.biolog.com/pdf/pmmlit/00P134rC%20PMM%20Broch%20PM-M1%20to%20-M14.pdf.

Medium MC-0 was used for CCH inoculated into PMMs 1–4, while complete medium was used for CCH inoculated into PMMs 5–8, 11, 13, and 14. CCH at a concentration of 1 × 10^4^ cells/well (PMMs 1–4; 50 *μ*L/well) or 2 × 10^4^ cells/well (PMMs 5–8, 11, 13, 14; 50 *μ*L/well) were seeded on two sets of microplates. The first one was used as the control set, where nontreated CCH were cultured. CCH seeded in the second set of plates were exposed to* M. synoviae* membranes. Both sets of PMs containing CCH were first incubated for 20 h to allow cells to catabolise all nutrients in medium MC-0. One set was subsequently inoculated with 15 *μ*L of* M. synoviae* membranes/well (protein concentration of approximately 1 mg/mL), while 15 *μ*L of PBS was added to each well in the control set of PMMs. After additional 5 h of incubation, 15 *μ*L of MB dye was added to each well and the PMMs were transferred to OmniLog incubator/reader (Biolog, US), where formazan production was recorded in 15-minute intervals for the next 24 h.

In order to test the effect of viable* M. synoviae* on CCH, the protocol was modified as follows. CCH were suspended in modified complete medium composed of IF-M1, supplemented with 0.3 mM glutamine, 1.87% FBS, and 0.63% chicken serum. Two sets of PMMs 6–8 were inoculated with 2 × 10^4^ cells/well (50 *μ*L/well) and incubated at 38°C in a 5% CO_2_ atmosphere for 30 h. CCH in one set of PMMs were then infected with 10 *μ*L of* M. synoviae* WVU 1853 culture (approximately 10^7^ CFU/well). To the control set of PMMs, 10 *μ*L of PBS/well was added. After additional 14 h of incubation, MB dye was added and the microplates were incubated in OmniLog that followed the kinetics of formazan production every 15 min for the next 24 h.

To exclude the influence of* M. synoviae* metabolism on dye reduction an additional control experiment was performed. Viable* M. synoviae* cells from the same broth culture that was used for CCH infection were seeded into microplates PMMs 6–8 at 10^7^ CFU/well. Microplates were incubated for 14 h, followed by addition of MB dye and further incubation. Kinetics of formazan production were assessed as described before.

### 2.6. Data Quantification and Statistical Analysis

Respiratory kinetics data was analysed using OmniLog programs File Management/Kinetic Analysis and Parameter Management. The level of CCH respiration in each well was normalized by subtracting the background signals detected in well A1, containing no supplements (only cells, medium and MB dye). For significance of respiratory activity, the cut-off value was set to 500 for metabolic plates and 5000 for sensitivity plates. In assessing the effect of* M. synoviae* or* M. synoviae* membranes, respiratory signals in each well were first normalized to the background by subtracting A1 and then compared to the normalized values of CCH respiratory signal in the corresponding wells. Differences in areas under corresponding graphs that were higher than 500 (metabolic plates) or 5000 (sensitivity plates) were used as criteria for significance of differences between the metabolic rates of CCH and CCH exposed to* M. synoviae *or its membranes. We have evaluated the effects on global metabolic activity in either treated or nontreated CCH by calculating the percentages of changes in respiration on all plates by dividing the number of positive results (all wells, where significant increase in respiration was detected by criteria described above) by the total number of substrate wells on all tested plates. To confirm statistical relevance of the differences in the metabolic activity or sensitivity, data from entire plates containing CCH were compared to corresponding plates containing CCH exposed to* M. synoviae* or its membranes using the Wilcoxon test for two paired groups. Nonnormal distribution of data was assumed. Differences between pairs of plates were considered significant for* P* values under 0.05.

## 3. Results

### 3.1. Data Quantification

**Figure 2:**
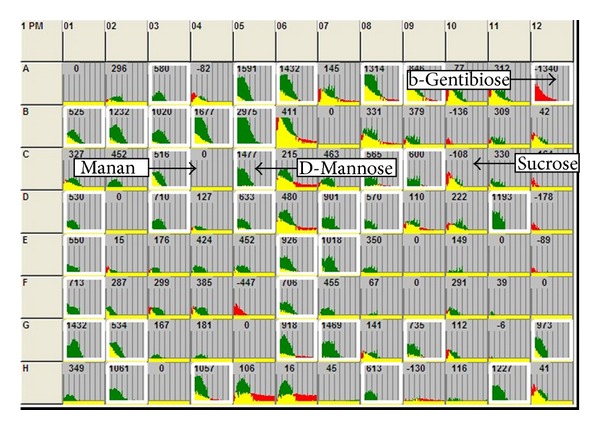
An example of the compared carbon source metabolic activities of CCH and CCH exposed to* M. synoviae* WVU 1853 membrane fraction (MSm) in PMM 1 containing different carbon and nitrogen sources. Kinetic data were collected using the Biolog Omnilog instrument and software. The curves show the time course (horizontal axis) of the amount of purple colour formed from tetrazolium dye reduction (vertical axis) in each of the 96 wells. All respiratory signals were previously corrected to the respiration of CCH in well A1, containing no supplements. Data from CCH are shown in red, from CCH+MSm in green, and yellow is the overlapping of the two kinetic curves. Wells where differences between respiratory signals reached a cutoff value (>500) are encircled white and are of two types: (i) C sources more rapidly metabolized by CCH compared to CCH+MSm are shown in red (A12, b-gentibiose); (ii) C sources more rapidly metabolized by CCH+MSm compared CCH are shown in green (C5, D-mannose). Wells that are not encircled represent compounds where metabolism was not detected or are metabolized equally both in CCH and CCH+MSm and are shown in yellow (C4, mannan; C10, sucrose).

In this study, CCH and CCH exposed to viable* M. synoviae* or its membrane fraction were assayed in 504 different conditions, that is, compounds, spotted in 1007 wells of 11 different PMMs. CCH respiration on metabolic and ion plates was considered to be a consequence of the ability of cells to metabolize a certain component as an energy/ion source. On the other hand, changes in respiration on sensitivity plates were considered to be a consequence of stress. The rates of respiration in each well of the PMM plates were monitored and recorded by the OmniLog instrument and analysed by the OmniLog-PMM software, which generated time course curves for respiration in each well. The outputs are colour coded kinetic graphs. When two experimental conditions were compared, one is shown in red, another in green, and the overlap in yellow. Hence, green shows more rapid metabolism for CCH exposed to* M. synoviae* or its membranes, red for CCH alone, and the yellow shows an equal level of respiration in exposed and nonexposed CCH. An example of such output analysis is shown in Figure 2.

To evaluate the influence of* M. synoviae* on the global metabolic activity of CCH, we analysed respiration kinetics in all 11 PMMs, where we compared the changes in respiration for CCH alone or CCH incubated with* M. synoviae* membranes. After 25 h of incubation respiration was significantly increased in 9.4% and 15.5% of all tested wells for CCH and CCH treated with* M. synoviae* membrane fraction, respectively. Additionally, we have also evaluated the changes in respiration of CCH infected with live* M. synoviae* on 3 hormone/immune plates. When CCH and* M. synoviae* exposed CCH were incubated for 44 h in hormone/immune PMMs, respiration was significantly increased in 11.6% and 63% of wells, respectively.

We have also evaluated the respiratory activity of* M. synoviae* alone on PMMs 6–8, but since bacteria could not utilize the MB dye, no respiratory activity was observed (Supplementary Figure A1) (Supplementary Material available online at: http://dx.doi.org/10.1155/2014/613730). The absence of respiratory activity in this control experiment excludes bacterial contribution to dye reduction in our study; therefore, the differences we observed were the consequence of the changes in the metabolism of CCH.

When the respiration data from each plate with CCH were compared and statistically evaluated to respiration data from corresponding plates with CCH exposed to* M. synoviae* or its membranes, the *P* values for each pair of plates indicated high statistical significance of differences. For metabolic plates PMMs 1–4, *P* values ranged from *P* < 0,0001 to *P* < 0,0314. For ion plates, all three hormone/immune plates, and all three cytotoxic plates, *P* values were <0,0001. For hormone/immune plates, where CCH alone or* M. synoviae* exposed CCH were incubated for 44 h, *P* values were from *P* < 0,0233 to *P* < 0,0001.

### 3.2. Metabolic and Sensitivity Profile of CCH

Metabolic and sensitivity profiles of CCH were obtained using 11 phenotype microarray plates, where cells were incubated for 25 h (CCH_25_), and 3 additional hormone/immune plates, where cells were incubated for 44 h (CCH_44_). CCH_25_ were able to metabolize 48 C/N sources (Table 1) and utilize 14 ions (Table 1). Interestingly, out of 69 cytotoxic chemicals, only rifaximin, carmofur, mercaptopurine, and perillyl alcohol increased the respiration of CCH.

Respiration of CCH_25_ cultured in hormone/immune plates was detected only in wells containing hydrocortisone, progesterone, aldosterone, and adenosine. Longer cultivation of CCH (44 h) increased the number of metabolically active wells and in addition to adenosine revealed another 20 hormones/immune mediators that induced metabolic activity of CCH_44_ (Table 2).

### 3.3. Effect of* M. synoviae *Membranes on Metabolic Activity of CCH

*M. synoviae* membranes were used to estimate if/how membrane components (and its potential antigens) influence the metabolism, ion uptake, and sensitivity of CCH_25_. By comparing CCH_25_ alone and CCH_25_ exposed to* M. synoviae* membranes, we found 18 carbon/nitrogen sources where the respiration levels were equal in membrane exposed and nonexposed CCH_25_ (Table 1, C/N sources, rows with + for CCH_25_ and = for CCH_25_+MSm). Exposure of CCH_25_ to* M. synoviae* membranes increased the utilization of glycogen, maltotriose, D-maltose, D-glucose-6-phosphate, a-D-glucose, chondroitin-6-sulfate, D,L-lactic acid, a-keto-glutaric acid, and 3 dipeptides (Table 1, C/N sources, rows with + for CCH_25_, and ↑ for CCH_25_+MSm). Analyses revealed additional 48 carbon energy/nitrogen sources that were uniquely used by membrane exposed CCH_25_ but were not utilized by CCH_25_ alone (Table 1, C/N sources, rows with − for CCH_25_, and = for CCH_25_+MSm). 18 dipeptides and b-gentiobiose were used by CCH_25_ but not by membrane exposed CCH_25_ (Table 1, C/N sources, rows with + for CCH_25_, and − for CCH_25_+MSm).

Stimulation of cells with* M. synoviae* membranes had a direct effect on the ion uptake in CCH since their respiration was detected only in wells containing lithium chloride, ferric chloride, and magnesium chloride (Table 1, cytotoxic/anticancer agents), while nonexposed CCH_25_ respired in wells with additional 11 ions. One explanation is that the presence of* M. synoviae* membranes disturbed the ion uptake in CCH. Alternatively, in the presence of bacterial membranes these ions could become toxic for the cells. When CCH were exposed to bacterial membranes on cytotoxic plates, we observed an increase in respiration in CCH for 29 cytotoxic compounds, where the respiratory rates were significantly higher than in CCH_25_. Higher respiration could be a consequence of increased cell sensitivity or, alternatively, bacterial membranes could help cells to improve their resistance to the toxic substances. Interestingly, respiratory signal that was detected in CCH_25_ exposed to carmofur or rifaximin ceased in CCH exposed to* M. synoviae* membranes (Table 1, cytotoxic/anticancer agents).

### 3.4. Effects of* M. synoviae* and Its Membranes on CCH Sensitivity to Hormones and Immune Mediators

Since* M. synoviae* is a chicken pathogen and can modulate host's immune system we wanted to see the phenotypic response of cells in the presence of various hormones and immune mediators. CCH exposed either to* M. synoviae* membranes for 25 h or to viable* M. synoviae* for 44 h were tested for their metabolic activity in the presence of 45 hormones and immune mediators (each spotted in 3–5 different concentrations in 270 wells) and compared to the metabolic activity of CCH alone in the same conditions. A control experiment was performed to exclude the influence of viable* M. synoviae* dye reduction on the overall value of measured CCH respiration (Supplementary Figure A1). It showed that* M. synoviae* do not metabolise MB dye in the experimental conditions used in the study and can be disregarded as a factor of influence.

In the 25 h incubation period, CCH respiration was detected only in 4 wells where hydrocortisone, progesterone, aldosterone, and adenosine were present. Longer incubation (44 h) made CCH_44_ sensitive to IL-2 and IL-8 at highest concentrations, IFN-*γ* at lowest, and TNF-*α* at intermediate concentrations. Sensitivity was also increased with additional 16 hormones (Table 2).

**Figure 3:**
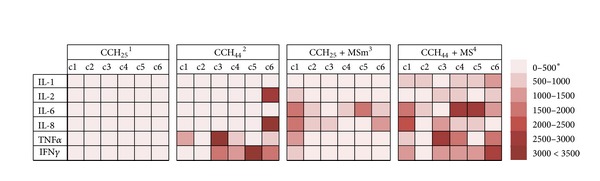
Sensitivity of CCH and CCH exposed to* M. synoviae* WVU 1853 whole cells or membranes as a function of cytokine concentration and time of exposure. ^1^ CCH_25_—respiratory activity of noninfected chicken chondrocytes exposed to cytokines for 25 h. ^2^ CCH_44_—respiratory activity of noninfected chicken chondrocytes exposed to cytokines for 44 h. ^3^MSm—*Mycoplasma synoviae* WVU 1853 membrane fraction. ^4^MS—*Mycoplasma synoviae* WVU 1853 live culture. *Different strength of respiratory activity signal is depicted in different shades of red.

Compared to the 4 hormones CCH_25_ responded to respiration of CCH_25_ exposed to* M. synoviae* membranes was detected with additional 13 hormones, IL-6, and lower concentrations of IL-2, IL-8, IFN-*γ*, and TNF-*α* (Table 2). Additionally,* M. synoviae* infected CCH_44_ became sensitive to another 19 hormones and to IL-1*β* and IL-6 as well as increased their sensitivity to IL-2, IL-8, IFN-*γ*, and TNF-*α* at all tested concentrations (Figure 3).

Sensitivity to certain compounds was shown to be treatment-dependent. Only CCH_44_ exposed to viable* M. synoviae* demonstrated sensitivity to calcitonin, calcitriol, creatine, dibutyryl-cAMP, gastrin, ghrelin, IGF-I, norepinephrine, PDGF-AB, and IL-1*β* while both membranes exposed CCH_25_ and* M. synoviae* exposed CCH_44_ (but not CCH alone) shared sensitivity to 4,5-a-dihydrotestosterone, *β*-estradiol, dexamethasone, hGH (somatotropin), FGF-1, thyroxine, IL-6, and insulin.

## 4. Discussion

The associations between joint inflammation and joint destruction are complex and not completely clarified both in humans and animal models. A complete approach would include the examination of numerous elements of complex interactions between the causative agent of inflammation, various immune cells, and immune mediators that are consequently present in the joint and the cells of joint tissues. Phenotype microarrays could prove useful for this purpose, as they allow screening of a large number of interactions using a simple and time saving approach.

These experiments were conducted in an attempt to characterize the effect of* M. synoviae* infection on the metabolic and sensitivity profile of CCH in the context of studying tissue destruction processes that occur in infectious synovitis. Using phenotype microarrays, we tested 504 compounds, obtained metabolic and sensitivity profiles of CCH, and demonstrated that they differ significantly from profiles of* M. synoviae* infected or membrane exposed CCH. For analysing changes in CCH carbon/nitrogen metabolism, use of live bacteria was avoided by exposing CCH to* M. synoviae* membranes. Although our control experiment showed that* M. synoviae* does not metabolise dye mix MB, their versatile profile of energy source utilisation [21] would affect the amount of carbon/nitrogen sources available for CCH.

Our results showed 48 carbon/nitrogen sources that were not metabolised by CCH alone but became acceptable for treated CCH, while the presence of bacterial compounds significantly increased uptake and respiration with 13 sources also used by CCH (Table 1). However, while the consumption of carbon/nitrogen sources increased after exposure to* M. synoviae* membranes, the opposite was shown for ions, where uptake ceased in most of ion wells (Table 1). The change in CCH metabolism after exposure to* M. synoviae* membranes could be attributed to the increased demand for energy used by the cell to adapt to the presence of membrane antigens and possibly to the lack of ions, some of which may bind to* M. synoviae* membranes. Changes in ion trafficking could be relevant to the biochemical background of oedema formation in joints of infected animals, which is most probably a direct consequence of inflammation-induced alterations in ion and water transport across the membrane of joint tissue cells and is currently not well explained even in humans [22].

The presence of* M. synoviae* membranes also induced an increased sensitivity to cytotoxic compounds tested in this study, from 4 in CCH alone to 29 after exposure (Table 1), including some with documented effect on chondrocytes, such as retinoic acid and colchicine [23, 24].* Mycoplasma* species are known for their apoptosis-modulating effects on host cells. However, these vary from apoptosis induction [25, 26] to inhibition of cell death [27, 28], indicating that additional research should be made to clarify these data.

From the aspect of clarifying the role of* M. synoviae* interaction with CCH in an environment of inflammation, we were especially interested in the results obtained on hormone/immune mediator PMMs. Comparing CCH_25_ to CCH_44_ revealed time-dependent effect of hormones and cytokines, resulting in an increase of the number of compounds eliciting a respiratory response from 4 to 20 (Table 2, Figure 3). Additionally, the response to IL-2, IL8, IFN-*γ*, and TNF-*α* that have a well-documented effect on human chondrocytes [10, 13, 14, 29, 30] was concentration-dependent (Figure 3).

As expected, presence of live* M. synoviae* or its membranes caused a significant change in the sensitivity profile of CCH (Table 2), with live bacteria causing a stronger response than membranes. This stronger effect of viable mycoplasma can partially be attributed also to longer incubation time in this experiment. Since our previous study [5] showed that* M. synoviae* can invade CCH cells and that the highest number of intracellular bacteria is present after 24 h, a longer incubation period was needed to evaluate the influence of both intracellular* M. synoviae* and those attached to the surface of CCH. Still, sensitivity to some compounds could be membrane-specific, since CCH infected with live bacteria responded to a higher number of compounds, but not all of the compounds were identical to those, when treated with membrane fraction (Table 2). Interestingly, certain compounds exhibited dual effect on infected/treated CCH, inducing an elevation or drop in respiration depending on the compound concentration and time of incubation (Table 2). Here, the ability of CCH to secrete certain cytokines could play a role, since presence of certain cytokines may induce secretion of others and cytokines act in an interdependent network. Although immune cells that are present in the inflamed joint are the key source of cytokines in arthritis, studies show human chondrocytes also secrete various cytokines and metabolic mediators [10, 31]. Information about the secretome of chicken chondrocytes is scarce. Although various cytokines including IL-1*β*, IL-18 nitric oxide, IFN*α*, and IFN*γ* have been found in the synovial fluid of infected chickens (Dusanic et al., unpublished observations) these are also secreted by activated immune cells including macrophages [7, 8]. Since cytokines act as a network, the potential ability of CCH to secrete certain cytokines could, in combination with the ones already present in wells of phenotype microarrays, influence the overall effect on chondrocytes, leading to a significant reduction in the expression of anabolic genes (e.g., aggrecan, collagen type II), upregulation of various catabolic genes (e.g., matrix degrading proteases) and a strong induction of intercellular mediators (e.g., leukemia inhibitory factor, IL-6). Activated chondrocytes are believed to additionally amplify the proinflammatory cascade of joint destruction through further release of cytokines (such as IL-1, IL-6, and TNF) [10, 31, 32]. Therefore, their role in the pathology of infectious synovitis should be considered.

Regulatory elements allowing* Mycoplasma* species to actively adapt to environmental signals are mainly unknown; phase and size variation of surface lipoproteins occur through distinct mutations and gene recombination and rearrangement. Still, several reports indicate that the hosts' immune elements and the presence of some nutrients could provide a selection pressure that drives surface antigen variation in* Mycoplasma, *allowing evasion of the hosts' immune response [33–37]. It would be interesting to see if* M. synoviae* infected CCH with changed metabolic pathways and products could also provide the selection pressure leading to changes in bacterial population phenotype. Similar reports have been published elsewhere [38–40].

This is the first report documenting the use of phenotype microarrays in the study of complex host-pathogen interactions. A significant difference between metabolic and sensitivity profiles of nonexposed and infected CCH was found in correlation with the presence of different carbon/nitrogen sources, ions, hormones, cytokines, and cytotoxic compounds. Although individual compounds and biochemical pathways were not analysed in detail, it is clear that the usefulness of this new technology in the area of host-pathogen research lies in the opportunity for high-through output screening of metabolic changes, as well as responsiveness of tissues to signals from activated immune cells, for instance, in the context of bacterial infection.

## 5. Conclusions

This is the first study of host-pathogen interactions using phenotype microarrays for eukaryotic cells. We report significant differences between the metabolic and sensitivity profiles of CCH and CCH treated with* Mycoplasma synoviae* or its membranes. Since many of the tested compounds can be found in inflamed joints, the results are relevant to the understanding of the pathology of infectious synovitis, especially considering that not much is known about the role of chondrocytes in tissue destruction occurring in infectious synovitis.

## Supplementary Material

Supplementary Figure A1: Respiratory activity of control *Mycoplasma synoviae* WVU 1853 strain, which was used for CCH infection, on PMMs 6-8.

## Figures and Tables

**Table 1:** Metabolic and sensitivity profiles of CCH and CCH exposed to *M. synoviae* WVU 1853 membrane fraction.

C/N sources	CCH_25_^1^	CCH_25_+MSm^2^	Cytotoxic/anti-cancer agents	CCH_25_^1^	CCH_25_+MSm^2^
2,3-butanediol	−	+	12,4′-demethylepipodophyllotoxin	−	+
Acetic acid	−	+	Acivicin	−	+
a-D-glucose	+	↑	Aclarubicin	−	+
a-D-glucose-1-phosphate	−	+	Aklavine hydrochloride	−	+
Adonitol	−	+	Beta-peltatin	−	+
a-Hydroxy-butyric acid	−	+	Carmofur	+	−
a-Keto-glutaric acid	+	↑	Celastrol	−	+
a-Methyl-D-glucoside	+	=	Cepharanthine	−	+
a-Methyl-D-mannoside	+	=	Chloroquine diphosphate	−	+
b-Gentiobiose	+	−	Colchicine	−	+
b-Methyl-D-galactoside	−	+	Cytosine-beta-d-arabinofuranoside	−	+
b-Methyl-D-glucoside	−	+	Dactinomycin	−	+
b-Methyl-D-xylopyranoside	−	+	Daunorubicin hydrochloride	−	+
Chondroitin-6-sulfate	+	↑	Doxorubicin hydrochloride	−	+
D,L-b-Hydroxy-butyric acid	−	+	Elaidylphosphocholine	−	+
D,L-Lactic acid	+	↑	Emetine	−	+
D-Arabinose	−	+	Gossypol	−	+
D-Cellobiose	+	=	Indole-3-carbinol	−	+
Dextrin	−	+	Mercaptopurine	+	↑
D-Fructose	−	+	Mitoxantrone hydrochloride	−	+
D-Fructose-6-phosphate	+	=	Nocodazole	−	+
D-Fucose	−	+	Perillyl alcohol	+	↑
D-Glucosaminic acid	−	+	Piceatannol	−	+
D-Glucose-6-phosphate	+	↑	Picropodophyllotoxin	−	+
D-Lactitol	−	+	Podofilox	−	+
D-Maltose	+	↑	Quercetin dihydrate	−	+
D-Mannitol	−	+	Quinacrine hydrochloride	−	+
D-Mannose	−	+	Rapamycin	−	+
D-Melezitose	−	+	Rifaximin	+	−
D-Raffinose	+	=	Rotenone	−	+
D-Tagatose	−	+	Vinblastine sulfate	−	+
D-Trehalose	+	=			
g-Amino-N-butyric acid	−	+	**Ions**		
g-Hydroxy-butyric acid	−	+	Ammonium chloride	+	−
Glycogen	+	↑	Ferric chloride	+	↑
Hexanoic acid	−	+	Iodine	+	−
Lactulose	−	+	Lithium chloride	+	=
L-Glucose	−	+	Magnesium chloride	+	+
L-Glutamine	−	+	NaCl	+	−
L-Rhamnose	−	+	Potassium chloride	+	−
Malitol	+	=	Potassium chromate	+	−
Maltotriose	+	↑	Sodium molybdate	+	−
Melibionic acid	−	+	Sodium nitrate	+	−
Meso-tartaric acid	−	+	Sodium nitrite	+	−
Mono-methylsuccinate	−	+	Sodium pyrophosphate	+	−
Myo-inositol	−	+	Sodium sulfate	+	−
N-Acetyl-b-D-mannosamine	−	+	Zinc chloride	+	−
N-Acetyl-D-glucosamine	−	+			
N-Acetylneuraminic acid	−	+			
Pectin	−	+			
Stachylose	−	+			
Succinamic acid	−	+			
Sucrose	+	=			
Tricarballylic acid	−	+			
18 dipeptides^3^	+	−			
10 dipeptides^4^	+	=			
3 dipeptides^5^	+	↑			
11 dipeptides^6^	−	+			

^1^Respiration levels for CCH incubated for 25 h (CCH_25_) were determined by comparing respiratory rates of CCH in each well of a plate to the corresponding A1 well that contained no supplements (+ respiration was higher than in A1).

^2^Respiration levels for CCH exposed to *M. synoviae* WVU 1853 membrane fraction (1 mg/mL, 15 *µ*L/well for 5 h before the measurement started) were determined by comparing respiratory rates measured during 24 h to those of CCH_25_.

^3^Dipeptides: Asn-Glu, Asp-Glu, Glu-Ala, Glu-Asp, Gln-Glu, Gly-Ala, Gly-Arg, Met-Trp, Phe-Ile, Phe-Val, Pro-Glu, Pro-Gln, Ser-Tyr, Thr-Pro, Thr-Ser, Trp-Val, Val-Phe, and Val-Ser.

^4^Dipeptides: Asp-Trp, Asp-Val, Glu-Ser, Glu-Tyr, Glu-Val, Gln-Gly, Tyr-Gln, Tyr-Glu, Tyr-Tyr, and Val-Lys.

^5^Dipeptides: Asp-Phe, Gln-Gln, and Gly-Asp.

^6^Dipeptides: Ala-Val, Arg-Trp, Arg-Tyr, Asp-Gly, Gly-Ser, Phe-Asp, Tyr-Val, Val-Ala, Val-Asn, Val-Gln, and Val-Gly.

Legend:

+: Respiration above cut-off value set to 500 (area under corresponding graph: for CCH compared to A1, for CCH+MSm compared to CCH).

−: Respiration lower than 500.

↑: Respiration of CCH+MSm higher than respiration of CCH in the presence of the same compound.

↓: Respiration of CCH+MSm lower than respiration of CCH in the presence of the same compound.

=: Respiration present in both CCH and CCH+MSm, difference not significant.

**Table 2:** Sensitivity of CCH and *M. synoviae* WVU 1853 infected CCH to hormones and immune mediators.

Hormone/immune mediator	CCH_25_^1^	CCH_44_^1^	CCH + MSm_25_^2^	CCH + MS_44_^3^
3-Isobutyl-1-Methylxanthine	−	+	−	+/=
4,5-a-Dihydrotestosterone	−	−	+	+
Adenosine	+	+	+	+/↑
Adrenocorticotropic Hormone Human (ACTH)	−	+	+/=	+/↑/=
Aldosterone	+	−	+	+
*β*-Estradiol	−	−	+	+
Caffeine	−	+	−	+/↑
Calcitonin	−	−	−	+
Calcitriol (1a,25-dihydroxyvitamin D3)	−	−	−	+
Chorionic Gonadotropin Human (HCG)	−	+	−	+/=/↓/↑
Creatine	−	−	−	+
Dexamethasone	−	−	+	+
Dibutyryl-cAMP	−	−	−	+
Epinephrine	−	+	−	+/↓
FGF-1 (aFGF)	−	−	+	+
Gastrin	−	−	−	+
Ghrelin	−	−	−	+
Gly-His-Lys acetate salt	−	+	+	+/↑
hGH (Somatotropin)	−	−	+	+
Hydrocortisone	+	−	+/=	+
IGF-I	−	−	−	+
Insulin	−	−	+	+
L-leucine	−	+	−	+/↑
Luteinizing Hormone (LH)	−	+	−	+/↓/↑
Luteinizing Hormone Releasing Hormone (LH-RH)	−	+	−	+/↑/↓
Norepinephrine	−	−	−	+
Parathyroid Hormone	−	+	−	+/=
PDGF-AB	−	−	−	+
Progesterone	+	−	+	+
Prolactin	−	+	−	+/↓/=/↑
Resistin	−	+	+	+/=
Thyrotropic Hormone (TSH)	−	+	+	+/↓
Thyrotropin Releasing Hormone acetate salt (TRH)	−	+	−	+/=
Thyroxine	−	−	+	+
Triiodothyronine	−	+	+	+/↑
Vasopressin	−	+	+	+/↓/=
IL-1*β*	−	−	−	+
IL-2	−	+	+	+/↓
IL-6	−	−	+	+
IL-8	−	+	+	+
TNF-α	−	+	+	+/↑
IFN-*γ*	−	+	+	+/=

^1^Respiration levels for CCH incubated for 25 h (CCH_25_) or 44 h (CCH_44_) were determined by comparing respiratory rates of CCH in each well of a plate to the corresponding A1 well that contained no supplements. (+ respiration was higher than in A1).

^2^Respiration levels for CCH exposed to *M. synoviae* WVU 1853 membrane fraction (1 mg/mL, 15 *µ*L/well for 5 h before the measurement started) were determined by comparing respiratory rates measured during 24 h to those of CCH_25_.

^3^Respiration levels for CCH infected with live *M. synoviae* WVU 1853 (10^7^ CFU/well for 14 h before the measurement started) were determined by comparing respiratory rates measured during 24 h to those of CCH_44_.

Legend:

+: Respiration above cut-off value set to 500 in at least one concentration of hormone/cytokine (area under corresponding graph: for CCH compared to A1, for CCH+MSm/CCH+MS compared to CCH).

−: Respiration lower than 500.

+/=; +/↑; +/↓: Respiration was detected and was, compared to respiration in CCH, equal, higher, or lower at different concentrations of hormones/cytokines.
