# Supplementary material for: Phenotypic Characterization of Mycoplasma synoviae Induced Changes in the Metabolic and Sensitivity Profile of In Vitro Infected Chicken Chondrocytes
Source: Biomed Res Int. 2014 Aug 26;2014:613730. doi: 10.1155/2014/613730 (PMC4160629; doi:10.1155/2014/613730)
Supplement: Supplementary file 1 — Supplementary Figure A1: Respiratory activity of control Mycoplasma synoviae WVU 1853 strain, which was used for CCH infection, on PMMs 6-8. [file 613730.f1.pdf]

Appendix:

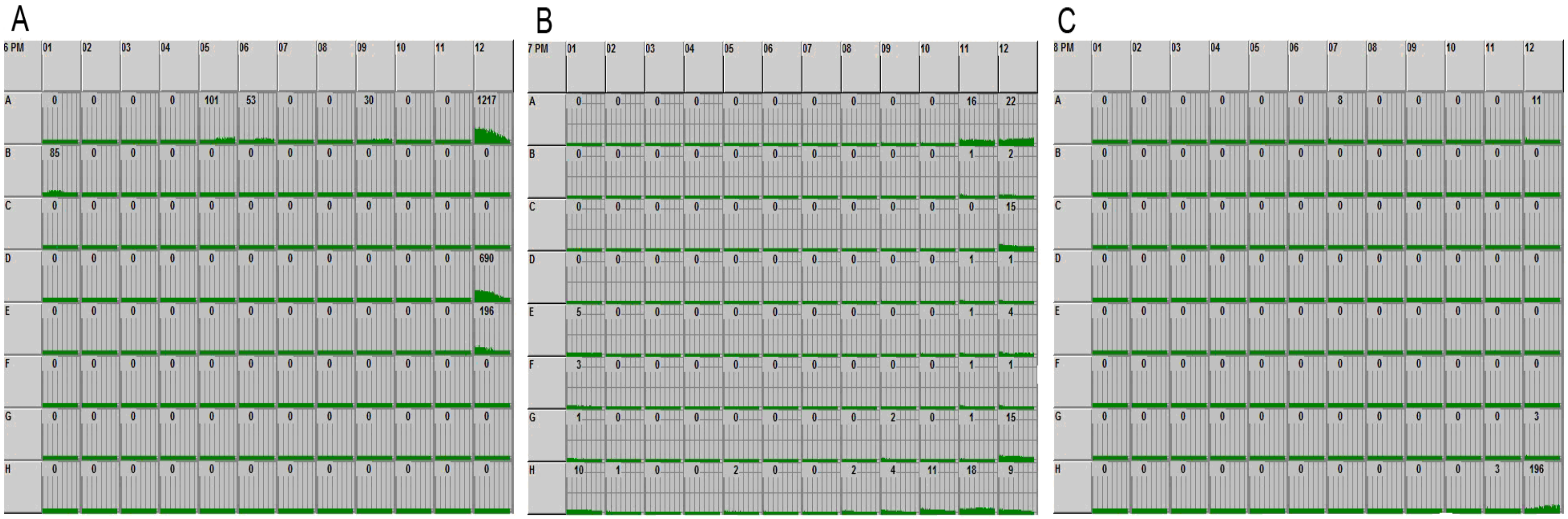

Supplementary Fig. A1: Respiratory activity of control *Mycoplasma synoviae* WVU 1853 strain, which was used for CCH infection, on PMMs 6-8. *M. synoviae* were plated in equal concentration as in the CCH infection experiment and incubated in the OmniLog incubator/reader together with plates containing infected and non-infected CCH. An equal amount of MB dye was added to all plates and the respiratory activity monitored as described for infected CCH. The absence of respiratory activity in this control experiment excludes bacterial influence on CCH metabolism in the study.
